# Longitudinal Blood DNA Methylation Changes During Weight-Loss Intervention and Dementia Progression Risk

**DOI:** 10.21203/rs.3.rs-10029889/v1

**Published:** 2026-06-17

**Authors:** David Lukacsovich, Juan I. Young, Lissette Gomez, Wei Zhang, Brian W. Kunkle, X. Steven Chen, Eden R. Martin, Lily Wang

**Affiliations:** University of Miami Miller School of Medicine; Dr. John T Macdonald Foundation, University of Miami, Miller School of Medicine; University of Miami Miller School of Medicine; University of Miami Miller School of Medicine; Dr. John T Macdonald Foundation, University of Miami, Miller School of Medicine; University of Miami Miller School of Medicine; Dr. John T Macdonald Foundation, University of Miami, Miller School of Medicine; University of Miami Miller School of Medicine

**Keywords:** DNA methylation, weight-loss, dementia, metabolic health, epigenome-wide association study

## Abstract

**Background:**

Weight loss improves metabolic health, but the DNA methylation (DNAm) changes induced by the lifestyle intervention and their relevance to dementia outcomes remain unclear. We studied longitudinal blood DNAm changes during an 18-month weight-loss intervention in the CENTRAL clinical trial and evaluated their relevance to dementia progression in an external cohort.

**Methods:**

We analyzed paired baseline and 18-month blood DNAm data from 103 male CENTRAL participants, including 47 in diet-only and 56 in diet plus physical activity groups. CpG-level methylation *M*-value changes between baseline and follow up were tested using linear mixed-effects models adjusted for dietary group, age, smoking score, leukocyte proportions, and random participant effects. Differentially methylated regions were identified using comb-p, and pathway enrichment was assessed using methylGSA software. Weight-loss-associated CpGs and regions were compared with cardiometabolic and dementia epigenome-wide association studies (EWAS) findings. In 117 Alzheimer’s Disease Neuroimaging Initiative participants with repeated DNAm and diagnostic follow-up, we tested whether DNAm changes at baseline resembling the reverse of the CENTRAL weight-loss profile predicted dementia progression.

**Results:**

At a 5% false discovery rate, 51 CpGs and three differentially methylated regions (DMRs) were identified in the diet-only analysis, and 49 CpGs and one DMR were identified in the diet plus physical activity analysis, that were significantly associated with the weight loss interventions. Enriched pathways included DNA double-strand break response and ATM-mediated phosphorylation in the diet-only analysis, as well as energy metabolism and insulin secretion in the diet plus physical activity analysis. Weight-loss-associated DNAm signals overlapped with cardiometabolic, inflammatory, and dementia-related EWAS findings in expected directions. In ADNI, 28 participants progressed clinically. Between-visit DNAm changes at baseline in converters showed higher correlation scores with the reversed diet-only DNAm profile than non-converters (mean correlation score, 0.131 vs. 0.037; *P*-value = 0.026), and higher correlation scores were associated with increased progression risk in a Cox model adjusted for age, sex, *APOE* ε4, baseline diagnosis, education, and smoking history (hazard ratio = 1.49 per standard deviation, *P*-value = 0.041). The diet plus physical activity profile showed a similar but weaker association.

**Conclusions:**

Weight-loss intervention was associated with blood DNAm changes enriched in genomic maintenance and metabolic pathways. External EWAS comparisons and ADNI validation suggest that weight-loss-responsive DNAm profiles may capture biological processes connecting lifestyle-related metabolic change with dementia progression risk.

## INTRODUCTION

Dementia, including Alzheimer’s disease (AD), is a major and growing public health challenge, and a substantial proportion of the cases may be prevented or delayed through modification of lifestyle and cardiometabolic risk factors^1^. Among these, obesity and related metabolic dysfunction are especially relevant because they contribute to vascular injury, chronic inflammation, insulin resistance, and other processes implicated in cognitive decline and AD^2^. Intentional weight loss and lifestyle improvement can improve metabolic health and may also benefit cognitive aging^3^, but the underlying biology through which these interventions might influence dementia-related risk remains incompletely understood.

DNA methylation (DNAm) is one of the most extensively studied epigenetic mechanisms. In contrast to genetic sequence, DNAm is responsive to environmental exposures and may reflect the biological impact of modifiable behaviors. In particular, it has been observed that blood DNAm differences are associated with habitual diet quality, dietary interventions, and cardiovascular health, with implicated loci enriched in cardiometabolic, inflammatory, and immune pathways^4–6^. Moreover, recent studies have also demonstrated DNAm is associated with incident dementia, AD neuropathology, and CSF AD biomarkers^7–9^. Together, these findings suggest that DNAm may help reveal the biological processes through which lifestyle exposures contribute to dementia biology.

The CENTRAL clinical trial evaluated dietary strategies based on Mediterranean/low-carbohydrate or low-fat diets, with participants subsequently assigned to physical activity or no physical activity^10^. This trial was conducted in a highly controlled workplace setting with on-site clinical, dietary, and intervention support, facilitating consistent intervention delivery and long-term follow-up. In the DNAm sub-study, fasting whole-blood samples were collected at baseline and 18 months from 120 participants, selected as 30 individuals from each intervention group with the greatest relative weight loss. This design provided an opportunity to examine within-person DNAm changes over the course of the intervention. The previous CENTRAL DNAm analysis identified blood DNAm signatures associated with successful weight-loss response^10^. Another CENTRAL analysis and additional caloric restriction intervention studies have similarly suggested that lifestyle modification can influence DNAm-based epigenetic clock measures of biological aging^11,12^.

While these previous studies provided encouraging evidence that the methylomes are responsive to lifestyle intervention, they do not yet clarify whether such changes are relevant to dementia. To address this gap, we performed a comprehensive analysis of the longitudinal CENTRAL DNAm data to identify intervention-related DNAm changes and assessed their potential relevance to biological pathways implicated in dementia. Importantly, using the external Alzheimer’s Disease Neuroimaging Initiative (ADNI) dataset, we further tested if the DNAm changes resembling the reverse of the CENTRAL weight-loss-associated profile were associated with increased risk of subsequent dementia progression.

## METHODS

### Study subjects

We analyzed DNAm data generated from fasting whole-blood samples of participants in the CENTRAL DNAm sub-study, an 18-month lifestyle intervention trial in which participants were assigned to either a Mediterranean/low-carbohydrate or low-fat dietary intervention, with or without physical activity^10^. For the ADNI validation study, we analyzed participants with blood DNAm samples available from at least two visits, as well as available diagnostic follow-up after the second DNAm visit. We additionally required participants to have the same diagnosis across their first two DNAm visits, either CN-CN or MCI-MCI.

### DNA methylation data

We implemented stringent quality control (QC) procedures to ensure reliable DNAm estimates. Supplementary Table 1 shows detailed probe and sample counts at each step. Samples were excluded if they showed poor bisulfite conversion (< 85%) or sex mismatch, and problematic probes were removed if they were cross-reactive^13^, located near SNPs, mapped to sex-chromosomes, or lacked annotation. Missing values and failed probes (detection *P*-values > 0.01) were imputed using the methyLImp2 R package^14^, which is designed specifically for methylation arrays. To reduce probe-design bias, we normalized beta values using β-mixture quantile normalization (BMIQ) as implemented in the wateRmelon R package^15^. Batch effects were corrected using the Harman R package^16^. Next, we performed principal component analysis (PCA) on the sample-by-CpG matrix (using methylation *M*-values). For each sample we computed Z-scores on the first two principal components (PC1 and PC2). We flagged a sample as an outlier if it exceeded ±3 standard deviations on either PC1 or PC2. To allow for assessment of within-person DNAm changes, we restricted the analyses to participants with DNAm data available at both baseline and 18 months. Also, because the DNAm sub-study included only a small number of female participants and sex-specific DNAm patterns could introduce additional heterogeneity, female samples were excluded. The ADNI validation samples were processed using the same pipeline, with an additional step of removing duplicated samples by retaining the sample with the higher bisulfite conversion rate (Supplementary Table 2).

### Statistical analyses to identify CpGs significantly associated with weight loss

To identify CpG sites associated with weight-loss intervention, we modeled longitudinal changes in DNAm between baseline and 18 months. DNAm levels were analyzed using methylation *M*-values, defined as the logit transformation of beta values, because *M*-values better satisfy linear-model assumptions (e.g., homoscedasticity)^17^. Smoking exposure scores were estimated using the EpiSmokEr R package, and immune cell proportions were estimated using the EpiDISH R package. Granulocyte proportions were calculated by summing the neutrophil and eosinophil proportions, as both are granular leukocytes.

All analyses were conducted separately for participants assigned to the diet-only intervention and those assigned to the diet plus physical activity intervention, using the same model in each group. For each CpG site, we fitted a linear mixed-effects model with methylation *M*-values as the outcome, fixed effects time (baseline, 18 months), diet (Mediterranean/low-carbohydrate, low-fat dietary), age, smoking score, estimated cell type proportions (CD4+ T cells, natural killer cells, B lymphocytes, monocytes, and granulocytes). In addition, random subject effects were also included to account for repeated DNAm measurements within individuals. Specifically, for each CpG, we applied the model:

$$M\text{-value}\sim \text{time}+\text{diet}+\text{age}+\text{smoking score}+\text{estimated cell-type proportions}+\left(1|\text{participant ID}\right)$$

The coefficient for time estimates the average within-person DNAm change from baseline to 18 months, adjusted for intervention arm, smoking, age, and blood cell composition.

To assess whether longitudinal DNAm changes differ by dietary intervention group, we fitted a mixed-effects model that additionally included a time-by-diet interaction term:

$$M\text{-value}\sim \text{time}+\text{diet}+\text{time}\times \text{diet}+\text{age}+\text{smoking score}+\text{estimated cell-type proportions}+\left(1|\text{participant ID}\right)$$

### Inflation assessment and correction

We estimated genomic inflation factors (lambda values) using the *bacon* method, which was specifically designed for EWAS^18^. The inflation factors (λ.bacon) were 1.22 and 1.18 for diet-only and diet + physical activity (diet + PA) analyses, respectively. The estimated biases were 0.41 for diet-only analysis and 0.30 for diet + PA analysis. After genomic correction using the bacon method^18^, the estimated bias were −2.25×10^−4^ and 9.16×10^−5^, the estimated inflation factors were λ.bacon = 1.00 and 1.00 for the diet only and diet + PA analyses, respectively. The bacon method was then used to compute bacon-corrected effect sizes, standard errors, and *P*-values for each dataset. To correct for multiple comparisons, we computed the false discovery rate (FDR). We considered CpGs with an FDR less than 5% after bacon correction to be statistically significant.

### Differentially methylated regions analysis

For region-based analysis, we used the comb-p method^19^, which scans genome-wide CpG locations and *P*-values to identify regions enriched with clusters of low *P*-values. We used *P*-values from each analysis (diet-only and diet + PA, analyzed separately) as input for comb-p and parameter settings with --seed 0.05 and --dist 750 (a *P*-value of 0.05 is required to start a region and extend the region if another *P*-value less than 0.05 was within 750 base pairs). These parameters were shown to have optimal statistical properties in our previous comprehensive assessment of the comb-p software^20^. To reduce false positives, we selected DMRs with Sidak-corrected *P*-values < 0.05 for multiple comparisons, at least three CpGs, and consistent direction of effect across all CpGs within the region.

### Functional annotation and pathway analysis

Significant individual CpG methylation signals and differentially methylated regions (DMRs) were annotated using gene annotations from Illumina and the Genomic Regions Enrichment of Annotations Tool (GREAT) software^21^. TSS-proximal promoter regions were defined as regions within 2 kb of the transcription start site. To identify biological pathways enriched with weight loss-associated DNAm, we used the methylRRA function from the methylGSA R package^22^, with single CpG *P*-values as input. Briefly, methylGSA computes a gene-wise value by aggregating *P*-values from multiple CpGs mapped to each gene, adjusts for the different numbers of CpGs per gene using Bonferroni correction, and then performs Gene Set Enrichment Analysis^23^ in pre-ranked mode to identify pathways enriched with significant CpGs. We analyzed pathways from the REACTOME database, and restricted analyses to pathways containing between 5 and 200 genes. To avoid gene sets where the enrichment signal was driven by only one or two genes, we required significant gene sets to include at least three genes in the “core enrichment” subset. Pathways with an FDR < 0.05 were considered statistically significant.

### Investigating the role of weight loss-associated DNAm in external cardiometabolic and dementia EWAS studies

To compare our results with previous EWAS findings, we searched for weight loss-associated CpGs (both significant individual CpGs and those located in DMRs) in the EWAS Catalog for CpG-trait associations in whole blood studies, including studies of BMI, obesity, body fat, waist circumference, waist-to-hip ratio, lipid traits, type 2 diabetes, C-reactive protein, physical activity, protein biomarkers and age^24^. In addition, to evaluate relevance to dementia, we also queried MIAMI-AD database^25^ (https://miami-ad.org/) using the CpG Query tool. For the phenotype input, we selected “AD Biomarker”, “AD Neuropathology”, “Dementia Clinical Diagnosis”, and “Mild Cognitive Impairment” and limited to external blood-based EWAS studies.

### Validation of CENTRAL-derived DNAm change profiles using external ADNI dataset

We evaluated whether DNAm changes opposite to those observed with weight loss in CENTRAL were associated with dementia progression in ADNI. CpGs were selected from the CENTRAL analyses based on nominal evidence of longitudinal DNAm change from baseline to 18 months (*P*-value < 0.05 for the time effect). For each selected CpG, the CENTRAL time-effect estimate was multiplied by −1 to generate an inverse CENTRAL weight-loss DNAm profile, referred to as the minus-estimate profile. This reversed vector represents the direction opposite to the CENTRAL weight-loss-associated DNAm change.

In ADNI, participants with two or more blood DNAm visits were selected, and those with the same diagnosis across their first two visits were included. Within-person DNAm change was calculated as visit 2 minus visit 1, referred to as the diff score. A subject-specific DNAm correlation score was computed as the Spearman correlation between each participant’s ADNI diff-score vector and the CENTRAL-derived minus-estimate vector across the selected CpGs. This correlation score was then standardized as a z-score.

The second ADNI DNAm visit was used as the baseline for progression follow-up. Progression was defined as CN-to-MCI/AD among participants who were CN at this baseline visit, or MCI-to-AD among participants who were MCI at this baseline visit. Follow-up time was calculated from the second DNAm visit to the first progression event or last available diagnosis. We compared DNAm correlation scores between converters and non-converters using Welch two sample t-test. In addition, associations between the standardized DNAm correlation score and progression were tested using Cox proportional hazards models adjusted for age, sex, *APOE* ε4 allele count, baseline diagnosis, education, and smoking history. This analysis workflow was applied to DNAm profiles derived separately from diet-only and diet + PA analyses.

Data used in the preparation of this article were obtained from the Alzheimer’s Disease Neuroimaging Initiative (ADNI) database (adni.loni.usc.edu). The ADNI was launched in 2003 as a public-private partnership, led by Principal Investigator Michael W. Weiner, MD. The primary goal of ADNI has been to test whether serial magnetic resonance imaging (MRI), positron emission tomography (PET), other biological markers, and clinical and neuropsychological assessment can be combined to measure the progression of mild cognitive impairment (MCI) and early Alzheimer’s disease (AD).

## RESULTS

### Study dataset

After quality control of DNAm samples, our analysis included data from a total of 103 male participants in the CENTRAL DNAm sub-study, including 47 in the diet-only group and 56 in the diet plus physical activity (diet + PA) group. Mean age was similar between these two groups: 47.2 ± 9.18 years in the diet-only group and 48.2 ± 9.62 years in the diet plus physical activity group. In the ADNI validation dataset (n = 117), the participants had a mean age of 75.7 ± 7.44 years; 50% (58 subjects) were women, and the mean duration of education was 16.3 ± 2.9 years (Supplementary Table 3). Approximately one third (36.8%, 43 subjects) carried at least one *APOE* ε4 allele, and 43.6% (51 subjects) reported a history of smoking. The participants were followed for 4.8 ± 3.0 years with 3 visits on average (Supplementary Table 3).

### DNA methylation at individual CpGs and DMRs are responsive to weight loss interventions

**Figure 1:**
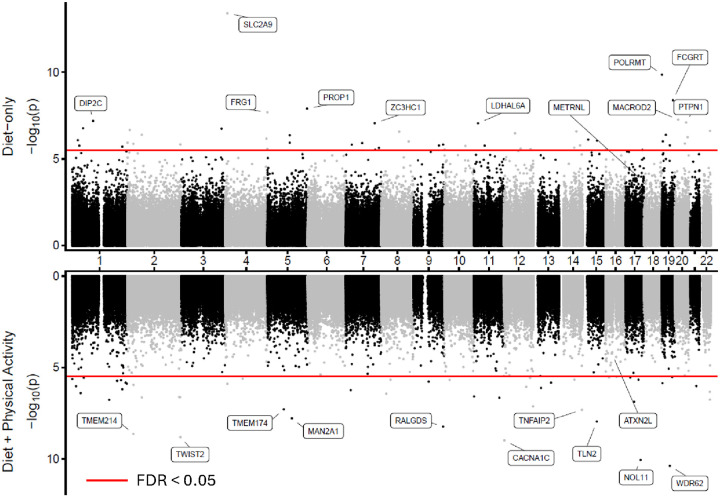
Miami plot of DNA methyiation changes associated with the diet-only and diet plus physical activity interventions. Shown are CpG-level associations with within-person DNAm changes over the 18-month intervention period. The upper panel shows results from the diet-only analysis, and the lower panel shows results from the diet plus physical activity analysis. Each point represents a CpG site ordered by chromosomal position. The y-axis shows −log10-transformed *P*-values. Red horizontal lines indicate the threshold corresponding to FDR < 0.05. Selected top signals are labeled by annotated genes.

Adjusting for dietary intervention group, age, smoking, and estimated cell-type proportions, our linear mixed-effects model identified 51 and 49 CpGs that changed significantly from baseline to 18-month follow-up in the diet-only and diet + physical activity (PA) analyses, respectively (FDR < 0.05; Figure 1, Supplementary Tables 4–5). In the diet-only analysis, most CpGs showed increased methylation during the intervention period, with 36 of 51 CpGs hypermethylated (71%) and 15 hypomethylated (29%). Approximately one third of these CpGs were located in TSS-proximal promoter regions (17/51 CpGs, 33%). In the diet + PA analysis, 40 of 49 CpGs were hypermethylated (82%) and 9 were hypomethylated (18%). A smaller proportion of CpGs in this analysis were located in TSS-proximal promoter regions (7/49 CpGs, 14%).

Across the two analyses, 94 unique CpGs were significant at FDR < 0.05. Effect estimates for these CpGs were positively correlated between the diet-only and diet + PA analyses (Spearman ρ = 0.504), indicating moderately consistent DNAm responses across the two intervention definitions. Six CpGs were significant in both analyses, and all showed consistent hypermethylation in both analyses (Table 1). These CpGs were mapped to gene bodies of the *RALGDS, C1orf95, WDR62, TMEM214*, and *SLC2A9* genes and the intergenic region.

Using CpG-level *P*-values as input, comb-p^19^ software identified three DMRs in the diet-only analysis and one DMR in the diet + PA analysis at a Sidak-adjusted *P* < 0.05 (Table 2). All four DMRs contained three CpGs. In the diet-only analysis, two DMRs located in intergenic regions, were hypermethylated, while one DMR, located in a CpG island at the *METRNL* promoter, was hypomethylated. In the diet + PA analysis, one DMR, located in a CpG island overlapping the *ATXN2L* promoter region, was hypomethylated.

### Pathway analysis

To further understand the biological processes underlying weight-loss interventions, we performed pathway analysis using the methylGSA software^22^, which uses continuous CpG-level *P*-values as input and does not rely on a predefined statistical significance threshold. In the diet-only intervention group, we identified three Reactome pathways significantly enriched with weight-loss-responsive DNAm (Supplementary Table 6). These pathways included *DNA Double-Strand Break (DSB) Response* and *Recruitment and ATM-mediated phosphorylation of repair and signaling proteins*, which are central to genomic maintenance. The accumulation of unrepaired DNA double-strand breaks is a well-documented feature of cellular senescence and has been implicated in aging-related neurodegenerative processes, including Alzheimer’s disease^26^. ATM kinase is not only a primary sensor of DNA damage but also plays important roles in neuronal genome maintenance, transcriptional regulation, and neuronal survival^27^.

In the diet plus physical activity group, we identified six Reactome pathways significantly enriched with weight-loss-responsive DNAm. Among these, *Integration of energy metabolism*, *Regulation of insulin secretion* pointed to metabolic processes that may be relevant to brain health. Cerebral glucose hypometabolism and brain insulin resistance are recognized metabolic features of AD and related neurodegenerative processes^28^. DNAm alterations in these pathways may reflect intervention-related changes in peripheral metabolic regulation, insulin sensitivity, and substrate utilization, with potential relevance to dementia risk.

### Comparison of weight loss-associated DNAm with external cardiometabolic and dementia EWAS studies

To assess whether weight loss-associated DNAm changes captured biologically relevant cardiometabolic signatures, we compared significant individual CpGs and CpGs located within DMRs identified in this study with prior EWAS findings from the EWAS Catalog^24^, which included CpG–trait associations reported at *P*-value < 1 × 10^−4^. In this comparison, we limited to CpG-trait associations whose directions were consistent with expected cardiometabolic changes following weight loss, such as lower BMI, improved glucose metabolism, and improved lipid profiles. For the diet-only intervention, our comparison identified 49 CpG-trait associations involving 18 unique CpGs (Supplementary Table 7). The overlapping traits included BMI, total cholesterol, HDL cholesterol, VLDL cholesterol esters, C-reactive protein, moderate physical activity, and incident type 2 diabetes, as well as circulating inflammatory and immune-related protein biomarkers. Several CpGs showed evidence of association with multiple cardiometabolic or inflammatory traits, including loci annotated to *DNAJC19/SOX2, ITGB4/RECQL5, NRXN3, CIT/PRKAB1*, and *C1orf95/ITPKB*.

The diet plus physical activity intervention showed a similar pattern, with 35 CpG-trait associations involving 15 unique CpGs in the EWAS Catalog comparison after applying the same directionality filter. The overlapping traits included BMI, waist circumference, fasting insulin, HDL cholesterol, incident type 2 diabetes, C-reactive protein, and multiple circulating protein biomarkers related to complement activation, inflammation, immune function, and metabolic regulation. Notably, a CpG annotated to *CD247* showed repeated overlap across BMI-related traits, waist circumference, C-reactive protein, fasting insulin, complement C3, and leucine-rich alpha-2-glycoprotein. Several additional CpGs, located near *C1orf95/ITPKB*, *ANK1*, and *RBM7/NNMT* also appeared across multiple cardiometabolic and inflammatory EWAS traits (Supplementary Table 8).

We next examined whether CENTRAL weight loss intervention-associated DNAm signals were also represented in external dementia-related EWAS studies using the MIAMI-AD database^25^. In this comparison, we restricted to CpG-dementia phenotype associations with nominal significance at *P*-value < 0.05 and in the expected direction, defined as opposite to the direction of DNAm change observed with weight loss in CENTRAL. For the diet-only intervention, 48 CpG-phenotype associations involving 27 unique CpGs were identified across dementia-related phenotypes. These included associations with AD, dementia, CSF total tau, and CSF phosphorylated tau in external blood-based EWAS (Supplementary Table 9). For the diet plus physical activity intervention, 55 CpG-phenotype associations involving 29 unique CpGs were identified (Supplementary Table 10). Overall, these external comparisons support the potential relevance of weight loss-associated DNAm signatures to cardiometabolic health and dementia-related biology.

### External validation of CENTRAL-derived DNAm profile correlation scores for dementia progression in ADNI

**Figure 2:**
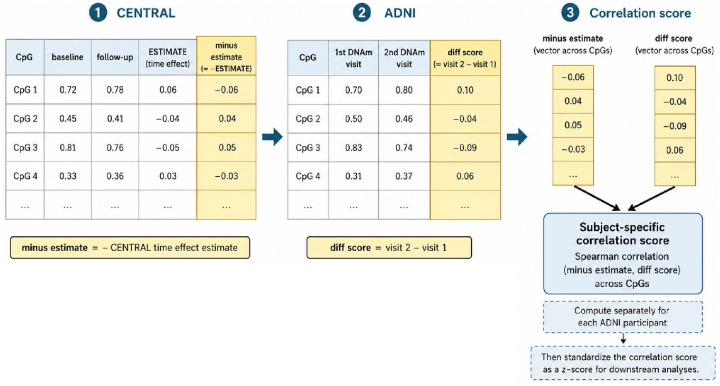
Computation of the DNAm correlation score in the ADNI validation analysis. The CENTRAL time-effect estimate for each selected CpG was multiplied by −1 to generate a reversed DNAm profile (“minus estimate”). In ADNI, within-person DNAm change at the same CpGs was calculated as visit 2 minus visit 1 (“diff score”). For each participant, the DNAm correlation score was computed as the Spearman correlation between the vectors of minus estimates and diff scores across CpGs with nominal evidence of longitudinal change in CENTRAL *(P* < 0.05 for the time effect), and then standardized as a z-score for downstream analyses. The reversal was used for interpretability so that the mean correlation scores were positive in both converters and non-converters.

We hypothesized that, if DNAm changes observed during CENTRAL weight loss reflect molecular changes related to healthier metabolic status, then DNAm changes in the opposite direction may be associated with increased risk of subsequent clinical progression. To this end, we tested whether participants whose longitudinal DNAm changes more closely resembled the reverse of the CENTRAL weight-loss-associated profile had increased risk of subsequent dementia progression. First, CpGs were selected separately from the CENTRAL diet-only and diet plus physical activity (diet + PA) analyses based on nominal evidence of longitudinal DNAm change (*P*-value < 0.05 for the time effect). Next, for each selected CpG, the CENTRAL time-effect estimate was multiplied by −1 to generate a reversed DNAm profile, referred to as the *minus estimate* (Figure 2). This profile represents DNAm change in the direction opposite to that observed during weight loss in CENTRAL. The reversal (i.e., *minus estimate*) was used for interpretability so that the mean correlation scores were positive in both converters and non-converters. Also, we used the nominal significance threshold to construct a broad genome-wide direction vector of CENTRAL longitudinal DNAm change, rather than a score driven only by the most statistically significant CpGs, since restricting the profile to FDR-significant CpGs would have left too few CpGs to compute a stable cross-CpG correlation score. The diet-only profile included 45,441 selected CpGs, and the diet + PA profile included 41,067 selected CpGs.

The ADNI validation analysis included 117 participants who remained clinically stable across their first two DNAm visits, defined as having the same diagnosis at both visits (CN-CN or MCI-MCI), and who had available diagnostic follow-up after the second DNAm visit. These participants included 58 females and 59 males. At the second DNAm visit, 52 participants were CN and 65 were MCI. During follow-up, 28 participants progressed and 89 did not progress; progression (i.e., conversion) events included 11 CN-to-MCI, 3 CN-to-AD, and 14 MCI-to-AD transitions. For each ADNI participant, within-person DNAm change at the selected CpGs was calculated as visit 2 minus visit 1, referred to as the *diff score* (Figure 2). We then computed a subject-specific DNAm correlation score as the Spearman correlation between two vectors across the selected CpGs: the CENTRAL-derived *minus estimates* and the ADNI *diff scores*. This correlation score was computed separately for each ADNI participant and then standardized as a z-score for downstream progression analyses.

**Figure 3:**
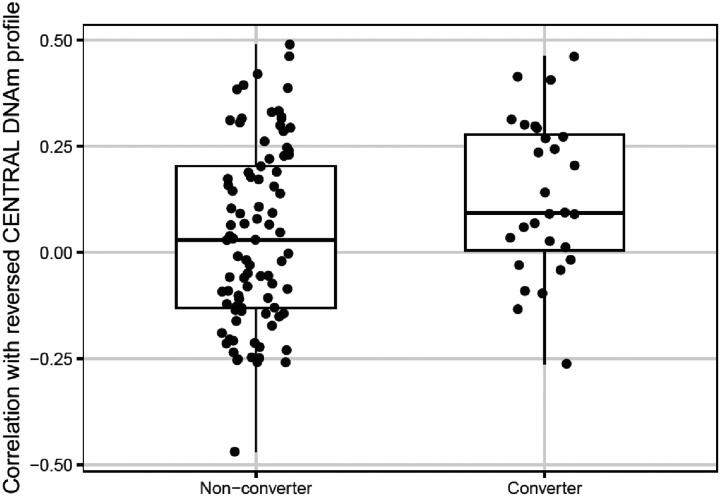
DNAm correlation scores by progression status in ADNI. The correlation score was calculated as the Spearman correlation between each participant’s within-person DNAm change across the first two DNAm visits in ADNI and the reversed CENTRAL ***diet-only*** DNAm profile (a weight-gain-associated DNAm profile). Higher values indicate greater alignment with the profile opposite to CENTRAL weight-loss-related DNAm changes. Each point represents one participant; boxes indicate the interquartile range, horizontal lines indicate the median, and whiskers represent values within 1.5 times the interquartile range.

**Figure 4:**
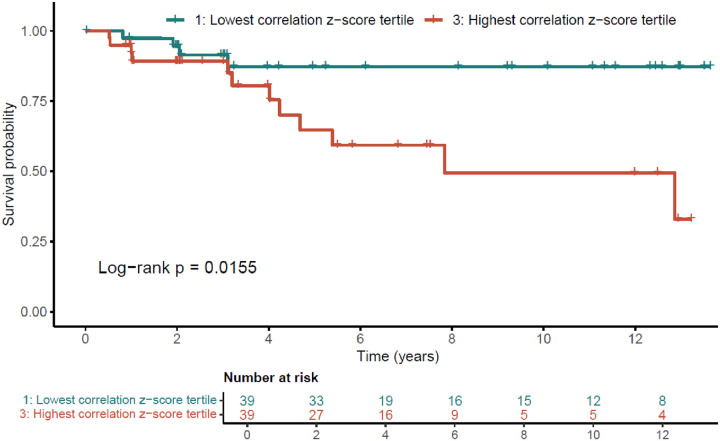
Kaplan-Meier curves comparing progression-free survival between ADNI participants in the lowest and highest tertiles of the standardized DNAm correlation score derived from the CENTRAL diet-only weight-loss profile. Higher scores indicate greater similarity between within-person ADNI DNAm change and the reversed CENTRAL profile. Participants in the highest tertile showed lower progression-free survival than those in the lowest tertile (log-rank test *P*-value = 0.0155).

Using the diet-only profile, participants who subsequently converted had higher DNAm correlation scores, indicating greater similarity between their ADNI diff-score vector during the clinically stable period and the CENTRAL-derived minus-estimate vector, than non-converters (Figure 3). The mean DNAm correlation score was 0.131 among converters and 0.037 among non-converters, with median scores of 0.093 and 0.030, respectively (*P*-value = 0.026). Kaplan–Meier analysis comparing the lowest and highest tertiles of the standardized correlation score showed that participants in the highest tertile had significantly lower progression-free survival and were more likely to progress than those in the lowest tertile (log-rank *P*-value = 0.015) (Figure 4). In the Cox proportional hazards model adjusted for age, sex, *APOE* ε4, diagnosis at the second DNAm visit, education, and smoking history, a higher diet-only DNAm correlation score was associated with increased risk of subsequent clinical progression (HR = 1.49 per standard deviation, *P*-value = 0.041; Table 3).

Results for the diet + PA-derived profile were directionally consistent but weaker. Converters had a higher mean DNAm correlation score than non-converters, 0.130 versus 0.025, with median scores of 0.150 versus 0.039, respectively (*P*-value = 0.043) (Supplementary Figure 1). Kaplan–Meier and adjusted Cox analyses both showed positive but nonsignificant associations with progression risk (log-rank *P*-value = 0.153; adjusted HR = 1.34 per standard deviation, *P*-value = 0.140; Supplementary Table 11, Supplementary Figure 2). Overall, these findings suggest that ADNI participants whose diff-score vectors during a clinically stable period more closely resembled the reverse CENTRAL weight-loss profile, especially the profile from diet-only analysis, had higher risk of subsequent clinical progression.

### Sensitivity analysis evaluating diet-by-time interaction

To evaluate whether longitudinal DNAm changes differed by dietary intervention group, we fitted mixed-effects models that additionally included a time × diet group interaction term. Across CpGs, there was little evidence that the longitudinal time effect differed by diet group. Only a small number of CpGs showed significant interaction effects: four CpGs in the diet-only analysis and one CpG in the diet + PA analysis (Supplementary Table 12). None of these CpGs overlapped with CpGs or DMRs significantly associated with weight loss in either analysis. These findings supported our use of the simpler primary main-effects model without the interaction term, which estimates the average DNAm change over time while adjusting for dietary intervention group.

## DISCUSSION

In this study, we analyzed longitudinal whole-blood DNAm data from the CENTRAL clinical trial to identify within-person methylation changes over 18 months of intentional weight-loss intervention and to evaluate their relevance to dementia. At a 5% FDR threshold, we identified 51 and 49 significant CpGs, as well as 3 and 1 DMRs, in the diet-only and diet + PA analyses, respectively (Table 2, Supplementary Tables 4–5). These DNAm signals were enriched in pathways related to genomic maintenance and metabolic regulation, overlapped with prior EWAS findings for cardiometabolic and inflammatory traits, and were also associated with dementia-related DNAm associations in MIAMI-AD (Supplementary Tables 7–10). Furthermore, in the external ADNI dataset, we found that participants whose within-person DNAm changes more closely resembled the reversed CENTRAL weight-loss profile, particularly the diet-only methylation change profile, had a higher risk of subsequent dementia progression. Together, these findings suggest that weight-loss-associated DNAm changes may capture biological processes connecting lifestyle-related metabolic change with dementia risk.

A review of recent literature revealed that a number of our top weight-loss associated CpGs and DMRs have been associated with cognitive health in prior studies. For example, in diet-only analysis, among genes with promoter regions associated with the most significant CpGs, the *POLRMT* gene encodes the critical enzyme responsible for mitochondrial DNA transcription and cellular energy production, which is essential for supporting the massive metabolic demands of the brain^29^. When this transcriptional machinery is disrupted, it triggers a neuronal bioenergetic collapse that can cause severe early-onset neurodevelopmental disorders, and may also contribute to the cognitive decline seen in AD^30^. Another notable gene is *FCGRT*, which regulates transport of immune proteins such as IgG. Prior work suggests that this pathway may contribute to amyloid-β clearance across the blood–brain barrier^31^. *PTPN1* is also relevant because it regulates insulin and leptin signaling. Since insulin resistance has been associated with cognitive decline and AD, methylation changes near *PTPN1* may reflect weight-loss-related changes in metabolic signaling that are relevant to brain health^32^. Finally, *LDHAL6A* is related to lactate metabolism, and lactate is increasingly recognized as an important energy source and signaling molecule in the brain^33^. The dysregulation of lactate-producing enzymes like *LDHAL6A* could disrupt the critical alternative supply of lactate to neurons, thereby exacerbating synaptic starvation and cognitive decline. Moreover, the *METRNL* gene associated with the DMR, is also biologically relevant. *METRNL* encodes a neurotrophic factor and adipokine that promotes neural growth, survival, and astrocyte function by regulating brain-derived neurotrophic factor (BDNF) levels in the hippocampus^34^. Recent studies suggest that reduced *METRNL* activity may worsen age-related cognitive impairment^35^.

Similarly, among the genes associated with top DNAm loci in the diet + PA analysis, the *CD247* gene encodes the CD3ζ chain, an immune signaling protein that also functions intrinsically within the central nervous system to regulate dendrite development, synaptic pruning, and the neural plasticity required for healthy cognition^36^. In the context of AD, altered *CD247* expression is increasingly recognized as a signature of the peripheral immune dysregulation and T-cell-mediated neuroinflammation that may contribute to synaptic loss^37^. Moreover, the DMR-associated gene *ATXN2L* encodes a critical regulator of RNA metabolism and cellular stress granule formation; notably, genome-wide association studies have implicated specific genetic variants of *ATXN2L* to enhanced cognitive resilience, verbal-numerical reasoning, and overall brain health in healthy populations^38^. Overall, these findings suggest that intentional weight loss may influence methylation near genes involved in mitochondrial energy production, immune transport, insulin signaling, metabolism and cellular stress response, all of which are biological processes relevant to dementia.

Consistent with these findings, several CENTRAL weight-loss-responsive CpGs also overlapped with prior blood EWAS associations for BMI, waist circumference, lipids, fasting insulin, type 2 diabetes, C-reactive protein, physical activity, and immune or inflammatory protein biomarkers. Moreover, in MIAMI-AD database, a subset of CENTRAL signals also showed associations with AD, dementia, CSF total tau, phosphorylated tau, or amyloid beta in directions opposite to those observed with weight loss. Because these comparisons were based on published EWAS results rather than *de novo* analyses of the external datasets, they should be interpreted as evidence of consistency with prior reports rather than formal replication.

A key strength of the present study is the external validation analysis in ADNI. We hypothesized that, if CENTRAL weight loss was associated with a DNAm profile potentially relevant to healthier metabolic change, then DNAm changes in the opposite direction might be associated with higher risk of clinical progression. To test this, we reversed the CENTRAL weight-loss effect vector and evaluated whether ADNI participants’ within-person DNAm changes were correlated with this reversed profile. Intriguingly, we found that ADNI participants whose longitudinal DNAm changes were more similar to the reversed CENTRAL diet-only profile had higher progression risk after adjustment for age, sex, *APOE* ε4 allele count, baseline diagnosis, education, and smoking history. Notably, this association was evident even though participants remained clinically stable during the interval over which DNAm change was measured, suggesting that the DNAm difference-based correlation score may capture molecular changes that precede subsequent clinical progression in dementia.

The diet + PA profile showed a consistent but weaker association with dementia risk. This may partly reflect differences in the number and composition of CpGs included in the two profile scores, as well as greater biological heterogeneity in the combined intervention. While the diet-only profile may capture a relatively coherent signature of weight-loss-related metabolic change, the diet + PA profile may combine methylation changes related to diet, adiposity, exercise adherence, fitness, inflammation, and musculoskeletal adaptation. Individual variability in exercise intensity, baseline fitness, and physiological response could further attenuate a blood-based aggregate score.

Several design and analytic features further strengthened this study. The CENTRAL DNAm data included matched baseline and 18-month samples, which enabled direct assessment of within-person methylation change. Baseline and follow-up samples were also balanced across methylation plates, reducing the risk that technical batch effects were confounded with time. The linear mixed-effects models accounted for repeated measures and adjusted for age, smoking score, and estimated major immune cell type proportions, reducing confounding from demographic, behavioral, and blood cell composition differences. Our sensitivity analysis showed little evidence of time-by-diet interaction, supporting the use of the primary main-effects model to estimate average longitudinal DNAm change while adjusting for dietary intervention group. In addition, the use of bacon correction, DMR analysis, pathway analysis, external EWAS comparisons, and validation in ADNI provided complementary evidence across CpG-, region-, pathway-, and profile-level analyses.

Several limitations should also be noted. First, the CENTRAL DNAm sub-study was relatively small and was enriched for participants who achieved substantial weight loss, which may limit generalizability to broader lifestyle-intervention populations, including individuals with limited weight-loss response. Second, our analysis was restricted to male participants because few women were included in the DNAm sub-study, which was conducted in a male-dominated workplace setting. This limits generalizability, particularly because sex differences in DNAm, body composition, metabolic response, and dementia risk may be important. Third, we did not have access to detailed person-specific weight-loss trajectories or adherence measures for all analyses, which limited our ability to distinguish methylation changes due to weight loss itself from those due to diet composition, physical activity, or other behavioral changes. Fourth, DNAm was measured in whole blood. Although blood is appropriate for scalable biomarker development, blood DNAm may not directly reflect methylation changes in adipose tissue, skeletal muscle, or brain, and estimated cell-type adjustment may not fully eliminate residual confounding from immune-cell heterogeneity. Finally, newer weight-loss treatments, including GLP-1 receptor agonists, may induce metabolic and inflammatory changes that differ from lifestyle-only interventions; future studies should evaluate whether similar or distinct DNAm profiles are observed in pharmacologic weight-loss settings.

In summary, this study provides evidence that intentional weight-loss interventions are associated with blood DNAm changes at individual CpGs and DMRs, with pathway-level enrichment in genomic maintenance and metabolic processes. External comparisons suggest their overlaps with cardiometabolic, inflammatory, and dementia-related methylation signatures, and validation in ADNI supports the potential relevance of these DNAm changes to subsequent clinical dementia progression. These findings support the development of DNAm-based molecular biomarkers for lifestyle intervention studies. Future work should replicate these findings in larger and more diverse intervention cohorts, integrate DNAm with gene expression and metabolic biomarkers, evaluate tissue specificity, and test whether nutritional patterns designed to support cognitive health, such as MIND-style dietary interventions, induce methylation profiles associated with reduced dementia risk.

## Supplementary Material

Supplementary Files

This is a list of supplementary files associated with this preprint. Click to download.

- ALLSUPPFIGURES6132026.pdf
- ALLSUPPTABLES6132026.xlsx
- Table123.docx

Tables 1 to 3 are available in the Supplementary Files section.

## Data Availability

The CENTRAL study dataset can be accessed at ArrayExpress repository https://www.ebi.ac.uk/arrayexpress/experiments/E-MTAB-8956. The ADNI datasets can be accessed at http://adni.loni.usc.edu. The scripts for the analysis performed in this study can be accessed at https://github.com/TransBioInfoLab/DNAm-weight-loss
